# Outpatient treatment with the wearable cardioverter defibrillator: clinical experience in two Dutch centres

**DOI:** 10.1007/s12471-017-0957-4

**Published:** 2017-02-10

**Authors:** A. F. B. E. Quast, V. F. van Dijk, A. A. M. Wilde, R. E. Knops, L. V. A. Boersma

**Affiliations:** 1 Heart Centre, Department of Clinical and Experimental Cardiology, Academic Medical Center, Amsterdam, The Netherlands; 2 Department of Cardiology, St Antonius Hospital, Nieuwegein, The Netherlands

**Keywords:** Wearable cardioverter defibrillator, Sudden death, Ventricular arrhythmias

## Abstract

**Introduction:**

The latest European Society of Cardiology Guidelines recommend consideration of a wearable cardioverter-defibrillator (WCD) for patients with a poor left ventricular ejection fraction (LVEF) who are at risk of sudden arrhythmic death but are not eligible for an implantable defibrillator. For these patients a WCD can be an alternative to long-term hospitalisation.

**Purpose:**

To evaluate the use of WCD therapy in these patient groups in two Dutch centres.

**Methods:**

All consecutive patients treated with the WCD between 2009 and 2016 were included from two centres in the Netherlands. Data on events and compliance were collected retrospectively through home monitoring systems and adjudicated by the investigators.

**Results:**

A total of 79 patients were treated with a WCD. Common indications were newly diagnosed cardiomyopathy without optimal medical treatment in 46 patients (58.2%) and bridge to implantable cardioverter-defibrillator (ICD) implant in 33 patients (41.8%). Bridge to implant indications consisted of contraindications for immediate implantation such as infections (e. g. previous device-related infections) and radiotherapy. Compliance was over 97% per day (median 23.3 h, 22.6–23.7), during a median of 79 days (50.0–109.8.0). Two patients (2.6%) received an appropriate shock (annual rate 13.6%), there was 1 (1.3%) inappropriate shock (annual rate 6.7%). In 24 patients (52.2%) without optimal medical treatment, the LVEF was sufficiently improved and ICD implant was avoided. Eight (10.1%) patients did not receive an ICD. In 45 patients an ICD was implanted (57.0%).

**Conclusion:**

WCD therapy provides a safe and effective treatment in outpatient setting for patients at high risk for sudden cardiac death and reduces the number of ICDs implanted.

## Introduction

An implantable cardioverter-defibrillator (ICD) is indicated in patients at risk for sudden cardiac death (SCD) due to ventricular arrhythmias, especially in patients with a reduced left ventricular ejection fraction (LVEF) [1]. Before an ICD indication can be established in patients with a de novo reduced LVEF, more than three months of optimal medical treatment (OMT) is required to allow improvement of the LVEF [2]. A time delay (of 40 days) is also pertinent for patients with recent myocardial infarction and/or revascularisation [3, 4]. The latest European Society of Cardiology (ESC) guidelines recommend class 2b consideration of the wearable cardioverter-defibrillator (WCD) in patients with a poor left ventricular (LV) function who are at risk of sudden arrhythmic death for a limited period, but are not candidates for an ICD [1]. Incidence of improvement of LVEF in newly diagnosed ischaemic and non-ischaemic cardiomyopathies is up to 41% [5]. Use of the WCD may therefore prevent non-evidence-based ICD implantations.

With a rapidly increasing population of ICD patients, the prevalence of ICD-related infections is increasing as well. In most cases, extraction of the device and leads is necessary. Recommendations of the Heart Rhythm Society state that the WCD can be an alternative to early re-implantation of an ICD when there is concern for ongoing infection [6]. Other indications for bridging to implant can be a current infection elsewhere, need for radiotherapy in the area of the ICD and a suspected arrhythmia disorder in which evaluation is still ongoing.

Continuous rhythm monitoring is provided by the WCD as well as detection of possibly fatal arrhythmias and automatic defibrillation of these arrhythmia episodes. Without the WCD these patients are bound to long-term hospitalisation for continuous rhythm monitoring to avoid SCD.

The effectiveness of the WCD was first described in 1998 [7]. Afterwards several large registries have proven the WCD to be a safe and effective treatment of episodes of potentially fatal arrhythmias [5, 8, 9]. The first use of the WCD in the Netherlands was described in 2011 [10]. A recent publication shows that WCD use in Europe is restricted and depends mainly on reimbursement [11]. The current study aims to describe the current use, number of prevented ICD implants and costs of WCD therapy in the Netherlands.

## Methods

All consecutive patients, from two large tertiary heart centres, who were treated with the WCD (Life Vest, ZOLL, Pittsburgh, PA) between 2009 and 2016, were included. Data on baseline characteristics, indication, duration of WCD wear and compliance, WCD therapy and adverse events were retrospectively collected through home monitoring systems and patient files.

Recorded episodes of arrhythmias were adjudicated by at least two investigators. Aborted therapy was defined as a detected episode for which the WCD was preparing to give therapy before this was manually aborted by the patient. Continuous variables are expressed as median, interquartile range (IQR) or mean ± standard deviation, depending on the distribution of the parameter.

For cost estimation, referential prices calculated by the Dutch Health Care Institute were used [12]. Specific costs for the cardiac outpatient follow-up visits were provided by the financial department of the Academic Medical Center, Amsterdam.

## Results

### Population

Between 2009 and 2016, 79 patients were treated with the WCD. All patients received their WCD within 24 h after the WCD indication was established and no other indication for hospitalisation remained. No patients were lost to follow-up; 61 patients were male (87.5%), median age was 54.0 (IQR 43.8–64.3) and the median baseline LVEF was 25% (IQR 18.0–39.3%). Seven patients (8.6%) were diagnosed with a genetic arrhythmia, 42 patients (51.9%) with non-ischaemic cardiomyopathy and 30 patients (37%) with ischaemic cardiomyopathy. One patient was suspected of Wolff-Parkinson-White syndrome and wore a WCD until definite diagnosis and ablation. One patient had non-sustained ventricular tachycardia (VT) and was suspected of cardiac involvement of myositis and 1 patient had VT originating in the area of an apical aneurysm after atrial septum defect closure (Table 1).

**Table 1:** Baseline characteristics

Characteristic	Patients (*n* = 79)
Male (*n*, %)	59 (74.9%)
Age, years (median, IQR)	54.0 (43.8–64.3)
*Genetic arrhythmia (n, %)* DPP6 mutation LQTS HCM (familial) iVF	*6 (7.6%)* 1 (14.3%) 1 (14.3%) 2 (28.6%) 2 (28.6%)
Ischaemic CMP (*n*, %)	29 (36.7%)
*Non-ischaemic CMP (n, %)* Dilated CMP Non-compaction CMP CMP eci Myocarditis Peri-partum CMP	*41 (51.9%)* 28 (68.3%) 2 (4.8%) 2 (4.9%) 7 (17.1%) 2 (4.9%)
Wolff-Parkinson-White syndrome (*n*, %) Other (*n*, %)	1 (1.3%) 2 (2.5%)
*Previous CIED implant (n, %)* One chamber TV-ICD Dual chamber TV-ICD S-ICD Pacemaker CRT-D	*26 (32.9%)* 2 (7.7%) 6 (23.1%) 6 (23.1%) 1 (3.8%) 11 (42.3%)
*Complication previous implant (n, %)* Infection	*23 (88.5%)* 23 (100%)
*Indication WCD (n, %)* Newly diagnosed iCMP Newly diagnosed non-iCMP Bridging to implant due to infection Bridging to implant due to other reason	*–* 12 (15.2%) 34 (43.0%) 23 (29.1%) 10 (11.9%)
*Ventricular arrhythmias (n, %)* Ventricular tachycardia Ventricular fibrillation Non-sustained ventricular tachycardia	*58 (73.4%)* 16 (27.6%) 10 (17.2%) 32 (55.2%)
*Supraventricular tachycardia (n, %)* Atrial fibrillation Atrial flutter Atrial tachycardia AV(*N*)RT	*16 (20.8%)* 13 (81.3%) 1 (6.3%) 1 (6.3%) 1 (6.3%)
LVEF, % (median, IRQ)	25.0 (18.0–39.3)

*AV(N)RT* atrioventricular (nodal) re-entry tachycardia, *CIED* cardio implantable electronic device, *CMP* cardiomyopathy, *iCMP* ischaemic CMP, *non-iCMP* non-ischaemic CMP, *DPP6* dipeptidylpeptidase 6, *HCM* hypertrophic cardiomyopathy, *IQR* interquartile range, *LQTS* long-QT syndrome, *LVEF* left ventricular ejection fraction, *S-ICD* subcutaneous implantable cardioverter-defibrillator, *TV-ICD* transvenous implantable cardioverter-defibrillator, *WCD* wearable cardioverter-defibrillator

The most common indication for WCD therapy was newly diagnosed cardiomyopathy, either ischaemic or non-ischaemic (46 patients, 58.2%). These patients were considered to be at high risk of ventricular arrhythmias, in most cases due to repetitive non-sustained VT or primary ventricular fibrillation (VF) at the time of myocardial infarction and a severely reduced LVEF. A total of 33 patients received WCD therapy as a bridge to ICD implant. Device-related infection occurred in 24 patients, 4 patients had an infection elsewhere, 2 patients had their ICD extracted in order to receive radiotherapy treatment, 2 patients needed to undergo cardiac surgery before implantation and 1 patient chose to wear a WCD while deciding if he wanted to proceed with ICD implantation after an episode of idiopathic VF.

Ventricular arrhythmias had been documented in 58 patients (73.4%), mostly non-sustained VT in 32 patients, sustained VT in 16 patients (27.6%) and VF in 10 patients (17.2%). Cardiac electronic implantable devices were previously implanted in 26 patients (32.9%), of which 8 transvenous ICDs, 6 subcutaneous ICDs (S-ICD), 1 pacemaker and 11 cardiac resynchronisation therapy-defibrillators (CRT-D). Of these 26 patients, 23 suffered a device-related infection, and all devices were extracted (Table 1). Two patients had an indication for device extraction due to radiation therapy, in 1 patient with a pacemaker the system was upgraded to an ICD.

### WCD compliance and therapy

Compliance was over 97% a day (median 23.3 h, IQR 22.6–23.7), during a median treatment duration of 79 days (IQR 50.0–109.8). Patients wearing the WCD as a bridge to implant used the device for a median of 79.0 days (IQR 54.8–102.3). In the patients with newly diagnosed cardiomyopathy this was 82.0 days (IQR 44.0–92.0).

Two patients (2.6%) received appropriate and successful therapy (calculated annual rate 13.6%) (Table 2). In 1 patient, the initial shock was successful. In the other patient a second shock was required to restore sinus rhythm. Both patients were previously treated with an ICD because of ischaemic cardiomyopathy.

**Table 2:** Results

Results	Patients (*n* = 79)
LVEF end WCD wear, % (median, IQR)	37.5 (23.5–45.8)
Number of days WCD wear (median, IQR)	79.0 (50.0–109.8)
Average hours WCD wear a day (median, IQR)	23.3 (22.6–23.7)
Appropriate therapy (*n*, %)	2 (2.6%)
Inappropriate therapy (*n*, %)	1 (1.3%)
Aborted therapy (*n*, %)	48 (63.2%)
*ICD implantation* One chamber TV-ICD Dual chamber TV-ICD S-ICD CRT-D	*45 (57.0%)* 7 (15.6%) 13 (28.9%) 8 (17.8%) 17 (37.7%)
Patients with recovered LVEF after WCD (*n*, %)	24 (30.4%)
*Patients with ICD indication that were not implanted (n, %)* Patient refused ICD Patient deceased before implantation Patient was implanted with LVAD	*8 (10.1%)* 3 (37.5%) 4 (50%) 1 (12.5%)
Hospitalisation days prevented in ‘bridge-to-implantation’ patients (median, IQR)	79.0 (54.8–102.3)

*IQR* interquartile range, *LVEF* left ventricular ejection fraction, *S-ICD* subcutaneous implantable cardioverter-defibrillator, *TV-ICD* transvenous implantable cardioverter-defibrillator, *WCD* wearable cardioverter-defibrillator

One patient (1.3%), diagnosed with ischaemic cardiomyopathy, experienced an inappropriate shock (calculated annual rate 6.7%). The mechanism was oversensing due to noise while putting on the vest. No malfunctioning of the device was observed; however, the patient received a new Lifevest. The patient experienced no disability after the inappropriate shock.

The home monitoring system recorded at least 1 episode of manually aborted therapy in 48 patients. Most cases were inappropriate sensing due to noise.

### ICD implantations

Of the 46 patients with a newly diagnosed cardiomyopathy, 24 (52.1%) had a sufficiently improved LVEF, therefore ICD implantation was not indicated at the time of final evaluation. Four of them had ischaemic cardiomyopathy with successful revascularisation (16.6%), and 19 had de novo non-ischaemic cardiomyopathy (79.2%). One patient (4.2%) used the WCD as bridge to implant. This last patient was not equipped with a permanent ICD because of an infection but had an improved LVEF at the end of the treatment for the infection (Table 2). One patient did not have an ICD indication after successful ablation of a malignant accessory bundle. Eight patients (10.1%) did not receive an ICD despite a clear indication. Three of them refused ICD implantation; 1 received a left ventricular assist device, and 4 patients died before planned ICD implantation (not due to arrhythmia). In 45 patients an ICD was implanted (57.0%) (Table 2).

### Mortality

Five patients (6%) died during treatment with the WCD. However, no arrhythmic deaths were observed. Two patients died of end-stage heart failure, the others from pneumonia, malignancy and type A dissection, respectively. In the 24 patients with improved LVEF during the use of WCD, who therefore did not receive an ICD, no ventricular arrhythmia or SCD was reported after cessation of WCD therapy, during a median follow-up of 1.6 years (IQR 0.1–3.3). Of the 3 patients who had refused ICD implantation despite a clear indication following current guidelines, 1 patient died at home from SCD.

### Costs

Costs for the use of the WCD in the Netherlands are 3000 €, charged per calendar month. After patients are discharged they are seen after a few days for a routine check-up. Further follow-up is done through home monitoring. Included in the calculated costs as presented in Table 3 are: WCD hire, check-up by pacemaker/ICD technician in outpatient clinic (41 €/visit) and 1 extra day of hospital admission during which the patient is instructed how to use the WCD (476 €/day). Costs of hospitalisation are calculated by using the average cost per day in a Dutch hospital on a nursing ward as calculated by the Dutch Health Care Institute. No additional costs were added in the calculation of costs for hospitalisation. The difference in calculated costs in favour of WCD ranges from 2671 to 23,327 € depending on duration of treatment (Table 3).

**Table 3:** Calculated estimation of costs of WCD wear compared with hospitalisation as bridge to implant for several time periods

	WCD in €	Hospitalisation in €	Difference in €
2 weeks	3993	6664	2671
4 weeks	3993	12,796	8.803
6 weeks	6993	19,194	12,201
8 weeks	6993	26,656	19,663
10 weeks	9993	33,320	23,327
12 weeks	9993	39,984	29,991

*WCD* wearable cardioverter-defibrillator

## Discussion

This study describes the largest Dutch cohort of WCD patients to date. Results show that for various indications, the WCD is a safe and effective treatment for ambulant monitoring and treatment of patients at risk of ventricular arrhythmia.

### Population

All patients in this cohort meet the specified criteria for which the latest ESC guidelines recommend WCD therapy. These results can therefore be considered representative for the population that is eligible for a WCD [1]. Compared with other WCD registries, baseline characteristics are similar [5, 9].

The utility of the WCD in patients with newly diagnosed non-ischaemic cardiomyopathy has been questioned by Singh et al. [13]. They concluded that the risk of appropriate therapy, and therefore SCD, in this particular subpopulation is minimal. On the other hand, specific patients could be classified as having a relatively high risk of ventricular arrhythmia, for example in patients with recurrent non-sustained VT or patients with incomplete revascularisation.

### WCD compliance and therapy

Patient compliance is excellent with a median wearing time of more than 23 h a day, which in addition to instruction and management is crucial for optimal protection. Median WCD wear in patients bridging to ICD implant in case of device-related infection in this cohort is longer than the period of treatment recommended by current guidelines. This can be explained by the fact that these patients are not hospitalised, therefore there is no need for minimal recovery time to re-implantation to reduce the period of hospitalisation. Outpatient therapy is cost-effective and gives a lower risk of hospital-acquired complications compared with long-term hospitalisation. Furthermore, it is associated with greater quality of life compared with inpatient treatment [14, 15].

No arrhythmic deaths were observed in this population, and all episodes of VT/VF were effectively treated by the WCD with an annual shock rate of 13.6%. As compared with the presented data, Singh reported a comparable incidence (1 shock per 7.8 patient-years). In the Wearit II trial, this was somewhat lower (5%). In a large cohort of post myocardial infarction patients treated with the WCD, the annual shock rate was calculated at 8.4% [16]. This comparison implies that the patients in the current trial are comparable with other WCD data and are truly at an elevated risk of ventricular arrhythmia.

One patient received an inappropriate shock due to noise oversensing. Noise oversensing is very common in the WCD, as can be seen by the large percentage of patients in which aborted therapy occurred (63.2%). The algorithms to prevent inappropriate shock together with the option to deactivate the device manually make the inappropriate shock rate low. Adequate patient instruction is crucial, as patients have to be able to prevent inappropriate therapy by deactivating the device manually. This might be considered a limitation of the device, as good instruction is mandatory and patients who are not willing or able to follow all the instructions should be precluded from WCD therapy.

### ICD implants

More than half of the 46 patients (52.1%) with a WCD as bridge to possible recovery in this cohort had a sufficiently improved LVEF (>35%) at time of final evaluation. This finding is in line with other registries [5, 8, 13] and supports the use of the WCD in obtaining a more accurate prophylactic ICD indication, with adequate protection in the early phase after diagnosis for specific patients who could be at specifically high risk of ventricular arrhythmia. This is expected to decrease the number of non-evidence-based ICD implants. Data from the United States showed that 22.5% of ICD implantations did not meet evidence-based criteria for implantation [17]. Most of these were implanted within 40 days of myocardial infarction, within three months of coronary artery bypass grafting or newly diagnosed heart failure without OMT [17].

### Costs

When considering WCD therapy, costs are of particular interest. In the Netherlands there is no well-defined reimbursement for this therapy. As mentioned before, WCD therapy is directly dependent on reimbursement [11]. Less than half of the responding centres in this survey confirmed the use of the WCD. Furthermore, 48% of these centres responded to have only used the WCD in <10 patients in the past year [11]. Costs can therefore be considered the major obstacle for physicians in using WCD therapy. Cost estimation in this study is meant to provide further insight into estimated costs of both treatment strategies.

WCD therapy could be perceived to be an expensive form of outpatient ICD treatment. However, our data show that during 12 weeks of use up to 30,000 € might actually be saved by using the WCD (Table 3). Nonetheless, there are important limitations to this calculation. Numerous factors are included in a complete cost calculation. Patients can suffer complications of either treatment and therefore require additional hospitalisation, emergency room visits, ambulance transportation or additional treatment. In addition to that, ICD re-implantation might be scheduled on a shorter term in a clinical setting than in case of outpatient treatment, but our estimation implies that there is an incremental gain in costs with longer treatment, in favour of the WCD. Cost-effectiveness of the WCD as a bridge to implant has previously been established by Healy et al. for patients with an infected cardioverter-defibrillator in the United States [18].

In patients with a newly diagnosed cardiomyopathy who are considered at high risk of SCD, bridging to recovery saves the costs of a possible non-evidenced based ICD implantation and lifetime follow-up, in case of recovery (in 52.1% in this study). On top of financial impact, the disadvantages of ICD therapy can be avoided in patients that do not meet evidence-based criteria.

## Conclusion

The WCD is a safe and effective treatment in an outpatient setting in patients in whom the indication for ICD is yet to be determined or device implantation is temporarily contraindicated. In patients with a newly diagnosed cardiomyopathy the number of non-evidence-based ICD implantations can be reduced with use of the WCD, as well as morbidity and reduction of quality of life due to long-term hospitalisation in patients with a bridge to implantation indication.
